# Ultra-high field upper extremity peripheral nerve and non-contrast enhanced vascular imaging

**DOI:** 10.1371/journal.pone.0175629

**Published:** 2017-06-29

**Authors:** Shailesh B. Raval, Cynthia A. Britton, Tiejun Zhao, Narayanan Krishnamurthy, Tales Santini, Vijay S. Gorantla, Tamer S. Ibrahim

**Affiliations:** 1 Department of Bioengineering, University of Pittsburgh, Pittsburgh, Pittsburgh, United States of America; 2 Department of Radiology, University of Pittsburgh, Pittsburgh, Pittsburgh, United States of America; 3 Siemens Medical Solutions, New York, United States of America; 4 Department of Plastic Surgery, Pittsburgh, Pittsburgh, United States of America; National Taiwan University, TAIWAN

## Abstract

**Objective:**

The purpose of this study was to explore the efficacy of Ultra-high field [UHF] 7 Tesla [T] MRI as compared to 3T MRI in non-contrast enhanced [nCE] imaging of structural anatomy in the elbow, forearm, and hand [upper extremity].

**Materials and method:**

A wide range of sequences including T1 weighted [T1] volumetric interpolate breath-hold exam [VIBE], T2 weighted [T2] double-echo steady state [DESS], susceptibility weighted imaging [SWI], time-of-flight [TOF], diffusion tensor imaging [DTI], and diffusion spectrum imaging [DSI] were optimized and incorporated with a radiofrequency [RF] coil system composed of a transverse electromagnetic [TEM] transmit coil combined with an 8-channel receive-only array for 7T upper extremity [UE] imaging. In addition, Siemens optimized protocol/sequences were used on a 3T scanner and the resulting images from T1 VIBE and T2 DESS were compared to that obtained at 7T qualitatively and quantitatively [SWI was only qualitatively compared]. DSI studio was utilized to identify nerves based on analysis of diffusion weighted derived fractional anisotropy images. Images of forearm vasculature were extracted using a paint grow manual segmentation method based on MIPAV [Medical Image Processing, Analysis, and Visualization].

**Results:**

High resolution and high quality signal-to-noise ratio [SNR] and contrast-to-noise ratio [CNR]—images of the hand, forearm, and elbow were acquired with nearly homogeneous 7T excitation. Measured [performed on the T1 VIBE and T2 DESS sequences] SNR and CNR values were almost doubled at 7T vs. 3T. Cartilage, synovial fluid and tendon structures could be seen with higher clarity in the 7T T1 and T2 weighted images. SWI allowed high resolution and better quality imaging of large and medium sized arteries and veins, capillary networks and arteriovenous anastomoses at 7T when compared to 3T. 7T diffusion weighted sequence [not performed at 3T] demonstrates that the forearm nerves are clearly delineated by fiber tractography. The proper digital palmar arteries and superficial palmar arch could also be clearly visualized using TOF nCE 7T MRI.

**Conclusion:**

Ultra-high resolution neurovascular imaging in upper extremities is possible at 7T without use of renal toxic intravenous contrast. 7T MRI can provide superior peripheral nerve [based on fiber anisotropy and diffusion coefficient parameters derived from diffusion tensor/spectrum imaging] and vascular [nCE MRA and vessel segmentation] imaging.

## Introduction

Ultra-high field [UHF] [≥ 7T] human magnetic resonance imaging [MRI] typically provides superior signal to noise ratio [SNR] and contrast to noise [CNR] ratios when compared to lower field [≤ 3T] MRI [1, 2]. Although, commercial [1.5T/3T] MRI is routinely used in upper extremity imaging [shoulder, forearm, hand, and wrist], the resolution achieved could be limited. UHF MRI could significantly improve the resolution capabilities of conventional MRI [3, 4]. However, 7T human MRI is fraught with technical challenges mainly related to radiofrequency [RF] field inhomogeneity and safety concerns for neurologic and whole-body imaging applications [1, 5–8],. The upper extremity offers the advantage of a compact isolated anatomy with relatively smaller electric size [when compared to the head/abdomen]. Therefore it can be relatively easier to generate uniform and safer RF field distributions for 7T in upper extremity imaging. As a result, significantly higher anatomical resolution and further improvements in contrast of tissue interfaces can potentially be achieved at 7T for upper extremity applications without increased scanning times and/or considerable compromise in overall image quality.

Higher SNR/CNR can be useful in providing intricate detail in neural [9], vascular [10], cartilage [11], tendon [12] and joint [13] imaging. As described in [14–16], 2D and 3D multi planar high resolution T1 weighted [T1] volumetric interpolated breath-hold exam [VIBE] imaging produces optimal anatomic detail and reveals high resolution structures. Nerves demonstrate a signal similar to adjacent soft tissue [14, 17]. High resolution T2 weighted [T2] double-echo steady state [DESS] can achieve excellent SNR/CNR for discriminating nerves from muscle, fascia, cartilage and synovial tissue in forearm and joint structures [18]. According to [19], susceptibility weighted imaging [SWI] is particularly useful for visualization of vasculature since it is highly sensitive to deoxyhemoglobin in venous blood, making it useful in imaging vascular trauma, abnormalities and visualizing neovascularization of tumors. Diffusion based sequences specifically monitor the random movement of water molecules in anisotropic tissue [20, 21].

One of the major obstacles in UHF MRI remains the limited availability of custom-designed coils [22] optimized for musculoskeletal applications. Some prior studies have demonstrated the utility and feasibility of customized coil designs for high-resolution wrist imaging at 7T [4, 23–25]. Our group has recently developed a custom-designed forearm/hand MRI RF coil system [26–28] for 7T UHF imaging. The combination of such a coil system in conjunction with 7T UHF MRI and many of the aforementioned sequences could bear significant potential in upper extremity imaging applications. Some of these applications include 1) sequential monitoring of regeneration after peripheral nerve [PN] repair, which is critical for evaluation of re-innervation and in planning treatment strategies [29, 30] and 2) monitoring vascular pathology or outcomes after vascular interventions without need for potentially nephrotoxic or anaphylotoxic contrast agents [31, 32].

In this study, utilizing a custom-designed RF coil system, we present results and findings from various MR sequences [T1 VIBE, T2 DESS, SWI, time-of-flight [TOF], diffusion tensor imaging [DTI], and diffusion spectrum imaging [DSI]] that were optimized for 7T upper extremity imaging while keeping scanning times similar to or lesser than those used at 3T. We show detailed SNR and CNR comparisons of 3T vs. 7T for T1 VIBE and T2 DESS sequences. Furthermore, we show nerve tractography [diffusion imaging], vessel segmentation [nCE magnetic resonance angiography], and imaging of cartilage, synovial fluid, bone marrow and joint anatomy in forearm, hand, and elbow at 7T.

## Materials and methods

### MR hardware and study participants

Briefly, a shielded design of an actively detuned transverse electromagnetic TEM resonator [26, 27] with two ports driven by a quadrature hybrid was used in this study. An in-house finite difference time domain [FDTD] package was utilized [6, 33] in order to calculate the B_1_^+^ field [transmit field responsible for excitation] and specific absorption rate [34] parameters. An eight channel receive-only insert array was designed and utilized with the TEM coil [26]. The array consisted of eight inductively decoupled surface loops [35] [each loop is 18x8 cm^2^ in size] distributed evenly to fit inside the structure of the transmit coil and cover the region of interest. The 7T RF coil system [Tx and Rx] covers 18 cm [in length] of anatomy [hand, forearm or elbow].

This prospective MRI study of upper extremity [forearm, hand, and elbow] was approved by University of Pittsburgh Investigational Review Board [IRB]. It was performed on three volunteers with appropriate written informed consent approved by above mentioned IRB protocol. Volunteers were screened to exclude those with musculoskeletal disease, upper extremity trauma, surgery, and/or comorbidities associated with musculoskeletal abnormalities [neuropathies, rheumatoid syndromes etc.]

### MR examination

Imaging was performed using a 7T whole-body MRI system [Magnetom, Siemens Healthcare, Erlangen, Germany] and 3T whole-body MRI system [Tim Trio, Siemens Healthcare, Erlangen, Germany] at University of Pittsburgh. The above mentioned RF coil system was utilized at 7T. [26–28]. A dedicated eight-channel extremity coil [Siemens, Cleveland, OH, USA] was used at 3T. Coils were positioned in the center of the magnet bore during all imaging sessions. Subjects were positioned prone within the coil with the hands [forearm, and elbow] placed over the head and immobilized with cushions, pads and sandbags to avoid discomfort.

### MR imaging

Five imaging sequences [T1 VIBE, T2 DESS, SWI, DTI, DSI, and TOF] were optimized by a highly experienced MR application scientist on the basis of a clinical MRI protocol for upper extremity pathologies. 3T and 7T protocols were optimized for same scanning time comparing a i] Higher Resolution [HR] protocol and ii] Lower Resolution [LR] protocol. We optimized the protocol on normal volunteers at 7T with representative varied resolutions using visual assessment for best image quality. Then for 3T, the protocol was adapted from the 7T protocol while using proper 3T imaging parameters. The protocol was again tested and optimized on normal volunteers and visual inspection was used to pick the best image quality. This study compares T1 VIBE, and T2 DESS qualitatively and quantitatively [SWI was only qualitatively compared.] 3T vs. 7T diffusion and TOF comparisons were not performed for this study. All the sequence parameters are documented in Tables 1 and 2. In the case of the 7T diffusion sequences, the specific b-values were optimized by balancing b-field directions—64—with higher b–values [DTI: 0 to 1300; DSI: 0 to 2000] while obtaining 1] smaller voxel resolution, and 2] acceptable time of acquisition [20] and SAR. 7T with parallel imaging factor of 2 and 3 were utilized to achieve the results shown [Tables 1 and 2].

**Table 1:** 7T and 3T T1VIBE, T2 DESS and T2* SWI forearm protocols for HR: Higher and LR: Lower resolution sequences. These sequences cover Figs 1, 2, 3, 4 and 6.

MR Sequences [Forearm]	T1 VIBE [Fig 1]		T2 DESS [Fig 2]		T2* SWI [Fig 4]	
**Field Strength**	**7T HR**	**3T HR**	**7T HR**	**3T HR**	**7T HR**	**3T HR**
TR/TE [ms]	12/4.49	12/5.21	18/5.22	18/5.22	23/15	23/14.2
Slice	288	288	238	288	128	128
FOV [mm^2^]	95x160	95x160	105 x 160	105 x 160	105 x 160	118 x180
Acquisition Matrix	277x512	277x512	270x448	270x448	302x512	302x512
Voxel Size [mm^3^]	0.34x0.31x0.30	0.34x0.31x0.30	0.39x0.36x0.40	0.39x0.36x0.40	0.35x0.31x0.80	0.39x0.35x0.80
FA	10	10	25	25	20	20
Acceleration factor	2	2	2	2	2	2
Bandwidth[hz/px]	150	150	169	169	119	119
Acquisition time [min]	5:34	5:34	4:43	4:43	6:08	6:08
**Field Strength**	**7T LR**	**3T LR**	**7T LR**	**3T LR**	**7T LR**	**3T LR**
TR/TE [ms]	12/4.47	12/5.14	18/5.22	18/5.22	23/15	23/14.2
Slice	288	288	176	240	128	128
Fov[mm^2^]	93x100	95x160	91 x 140	105 x 160	118 x180	118 x 180
Acquisition Matrix	273x320	191x352	193 x 320	232 x 384	227 x 384	227 x 384
Voxel Size [mm^3^]	0.34x0.31x0.41	0.50x0.45x0.30	0.47x0.44x0.50	0.45x0.42x0.40	0.52x0.47x0.80	0.52x0.47x0.80
FA	10	10	25	25	20	20
Acceleration factor	2	2	2	2	2	2
Bandwidth[hz/px]	150	150	195	194	119	119
Acquisition time [min]	4:36	4:01	3:36	4:08	4:42	4:42

**Table 2:** 7T in-vivo imaging protocols for elbow, forearm, and hand [Figs 3 and 5–9; T1VIBE, T2 DESS, T2* SWI, DTI, and TOF].

MR Sequences	T1 VIBE [Elbow]	T2 DESS [Elbow]	DTI [Forearm, Dir:64, b = 0,1300]	DSI [Forearm, b = 0 to 2000]	TOF [Hand]
Orientation	Coronal	Coronal	Axial	Axial	A/S/C
FOV [mm^2^]	93 x 100	91 x 140	70 x 62	70 x 62	85 x 208
Acquisition matrix	273 x 320	193x320	490x434	490x434	236x640
TR [ms]	12	18	7000	8000	12
TE [ms]	4.49	5.22	83	80	4.5
Slices	240	176	65	65	254 [single slab]
Bandwidth	150	195	-	-	163
Acquisition time	5:34	3:36	27	45	-
FA	10	25	180	90	19
Acceleration factor	2	2	2	2	3
Voxel Size [mm^3^]	0.34x0.31x0.41	0.47x0.44x0.5	0.14x0.14x3	0.14x0.14x1.70	0.36x0.33x0.40

### MR data analysis

#### Quantitative analysis

**Fig 1:**
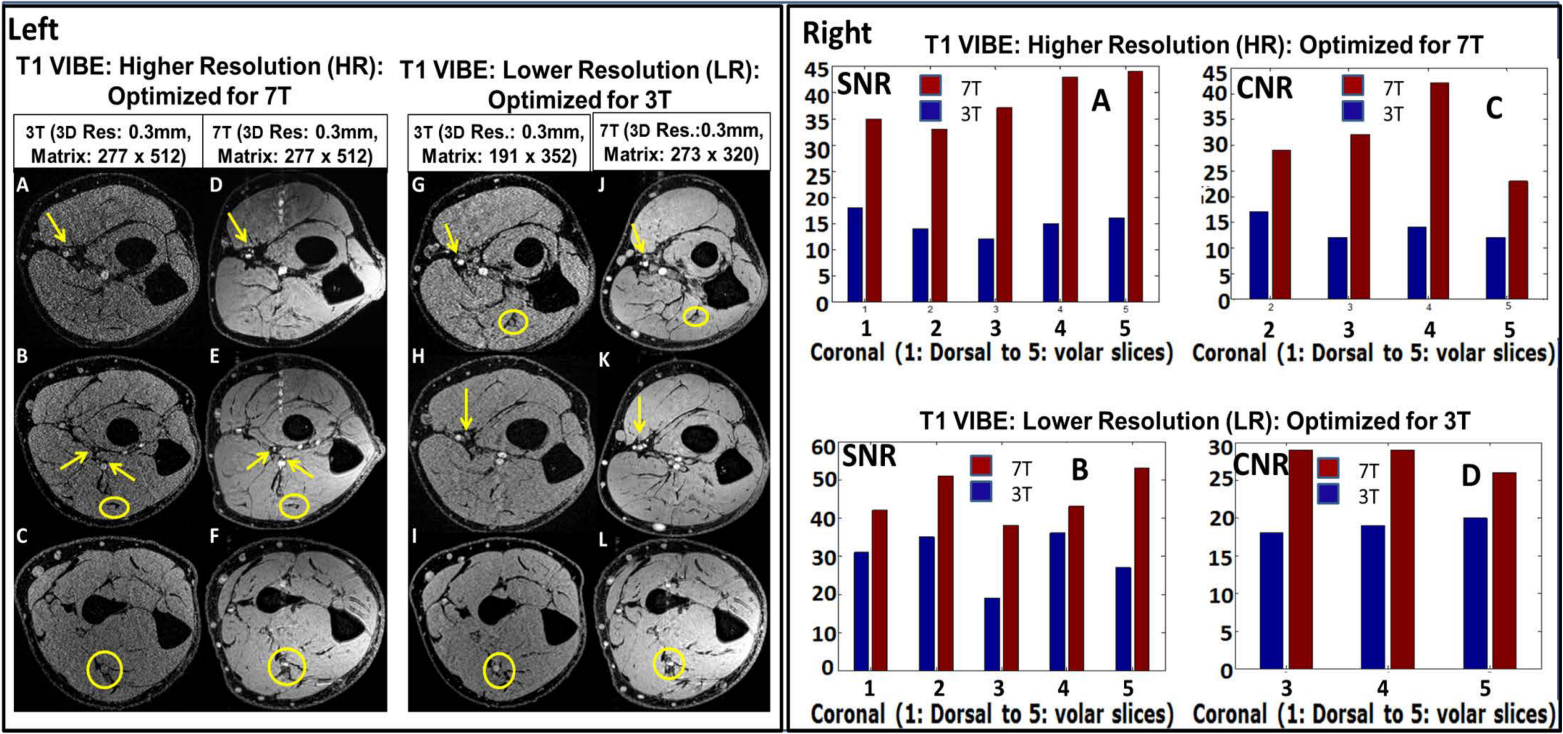
Left: 3T vs. 7T T1 VIBE images on both higher [HR which is optimized for 7T] and lower [LR which is optimized for 3T] resolutions [as described Table 1]. In the 3T images [A, B, C, G, H and I], smaller order vessels as indicated by arrows are barely visible; while they are well detected on the 7T images [D, E, F, J, K and L]. Yellow arrows indicate micro-vessel branches and yellow ellipses delineate nerves from surrounding muscle plane. Note motion and pulsatile flow artifacts [band of bright points] originating from the median artery in the anterior to posterior phase encoding direction. Right: SNR and CNR charts for T1 VIBE images. [A and B] and [C and D] represent SNR and CNR; respectively. The 7T SNR and CNR were ~ 2/1.5 times that of 3T for the HR/LR scans, respectively. Out of all three volunteer, the above data is from single volunteer only.

Quantitative 3T and 7T SNR and CNR were measured for T1 VIBE, and T2 DESS sequences over the complete volume of forearm [presented in Figs 1 [right] and 2 [right]]. Specifically, SNR was measured by calculating average signal intensity over a selected circular region [approximately encapsulates the forearm area excluding the skin] in each slice divided by mean standard deviation [SD] of the background noise going from slice 1 [starting from the dorsal aspect] to slice 5 [close to volar aspect]. The CNR was defined as the difference in mean signal intensity between bright tissue [vessel] and adjacent bone signal [and /or background noise]. Two musculoskeletal radiologists with over thirty-five years of combined clinical experience blindly evaluated images and sequences. All measurements were performed by the same researcher and verified by the MR application scientist and both musculoskeletal radiologists.

**Fig 2:**
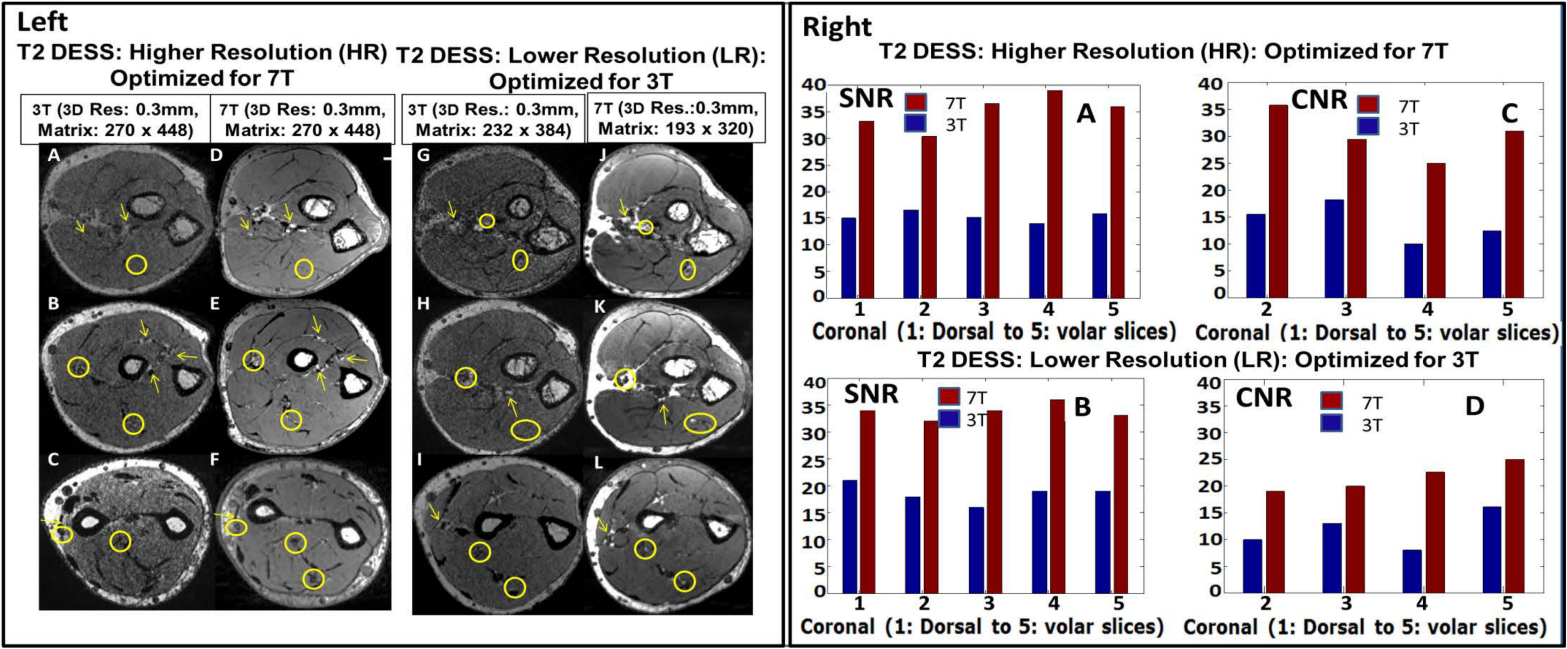
Left: 3T vs. 7T T2 DESS images on both higher [HR which is optimized for 7T] and lower [LR which is optimized for 3T] resolutions [as described Table 1]. Small order vessel [yellow arrows] and nerve [yellow circles] signals as indicated by circles are difficult to identify on the 3T [A, B, C, G, H and I] images but are well delineated on the 7T [D, E, F, J, K, L] images Right: SNR and CNR charts for T1 VIBE images. [A and B] and [C and D] represent SNR and CNR; respectively. The 7T SNR and CNR were ~ 2/1.5 times that of 3T for the HR/LR scans, respectively. Out of all three volunteer, the above data is from single volunteer only.

#### Qualitative analysis

Criteria for Evaluation of Image Quality: Contrast resolution, sharpness and clarity were the criteria considered in evaluation of image quality, using a scale as follows: 1-nondiagnostic, 2- poor, 3-fair, 4-good and 5-excellent [Table 3].

**Table 3:** 3T/7T MRI Analysis [Image Quality Scale: 1-nondiagnostic, 2- poor, 3-fair, 4-good and 5-excellent; Artifacts scale: 1-absent, 2-present but not affecting anatomic detail and 3-present and severely affecting image interpretation].

Qualitative Analysis	Image Quality factor		Artifact factor	
Field Strength	3T	7T	3T	7T
T1 VIBE Lower Resolution [Fig 1-Left]	3	3.5	1	1.5
T1 VIBE Higher Resolution [Fig 1-Left]	3.5	4	2	2
T2 DESS Lower Resolution [Fig 2-Left]	2.5	3	2.5	1.5
T2 DESS Higher Resolution [Fig 2-Left]	2	3	1	1
T2* SWI Lower Resolution [Fig 4]	1.5	2	2.5	2.5
T2* SWI Higher Resolution [Fig 4]	1.5	2	3	2.5

Evaluation of Imaging Artifacts: Presence of chemical shift, susceptibility artifact and motion artifact were considered in evaluation of artifacts using a scale as follows: 1-absent, 2-present but not affecting anatomic detail and 3-present and severely affecting image interpretation.

Evaluation of image quality and artifacts were both carried out for 3T and 7T for T1 VIBE, T2 DESS, and T2* SWI images [Figs 1 [left], 2 [left], 3–6]. The sequences were evaluated retrospectively and independently by two musculoskeletal radiologists who were blinded to the imaging parameters.

**Fig 3:**
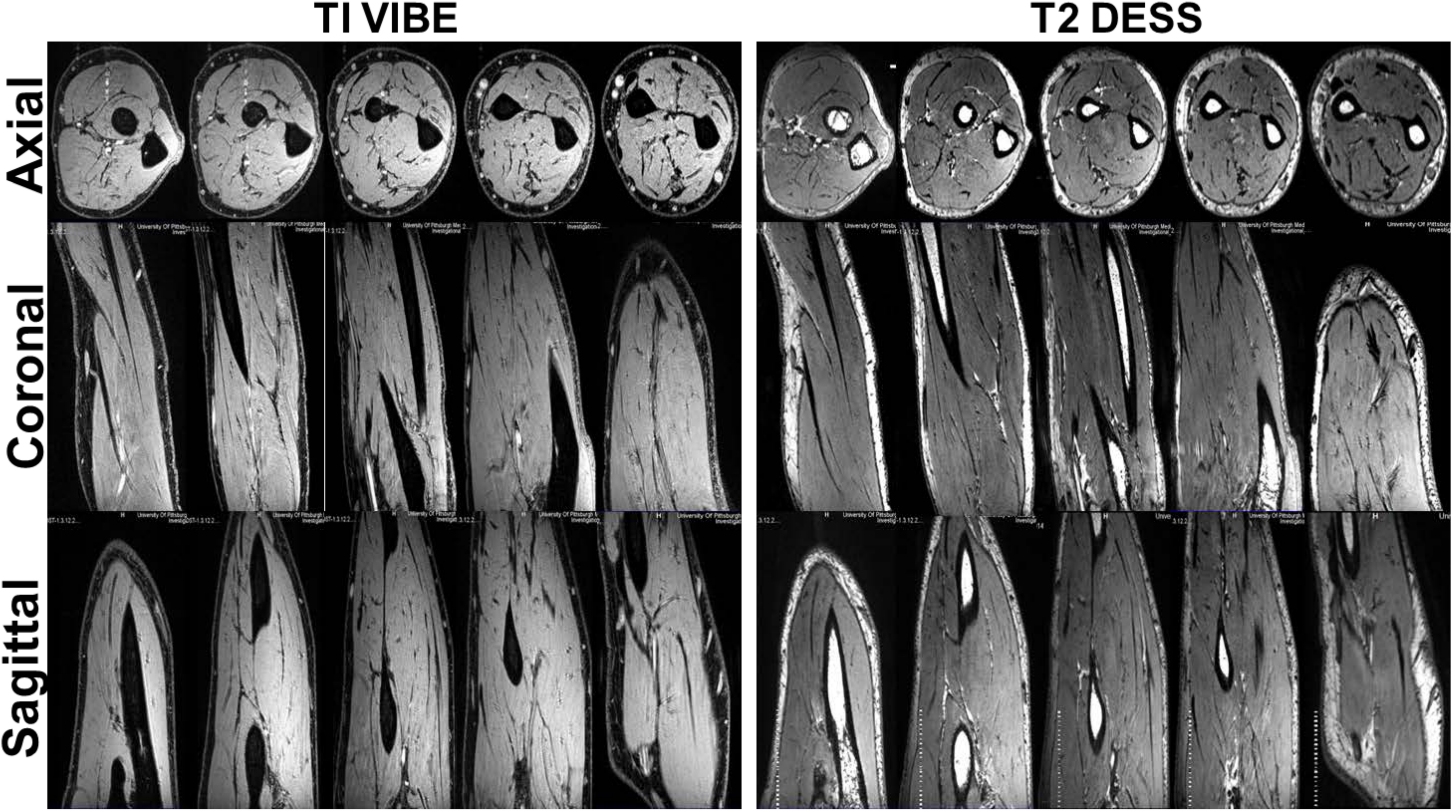
7T T1 VIBE and T2 DESS images of the forearm demonstrating homogeneous excitation in axial [elbow to wrist], coronal and sagittal planes. The B1+ field coefficient of variation was calculated to be 21% in the forearm [encapsulated within the coil volume].

**Fig 4:**
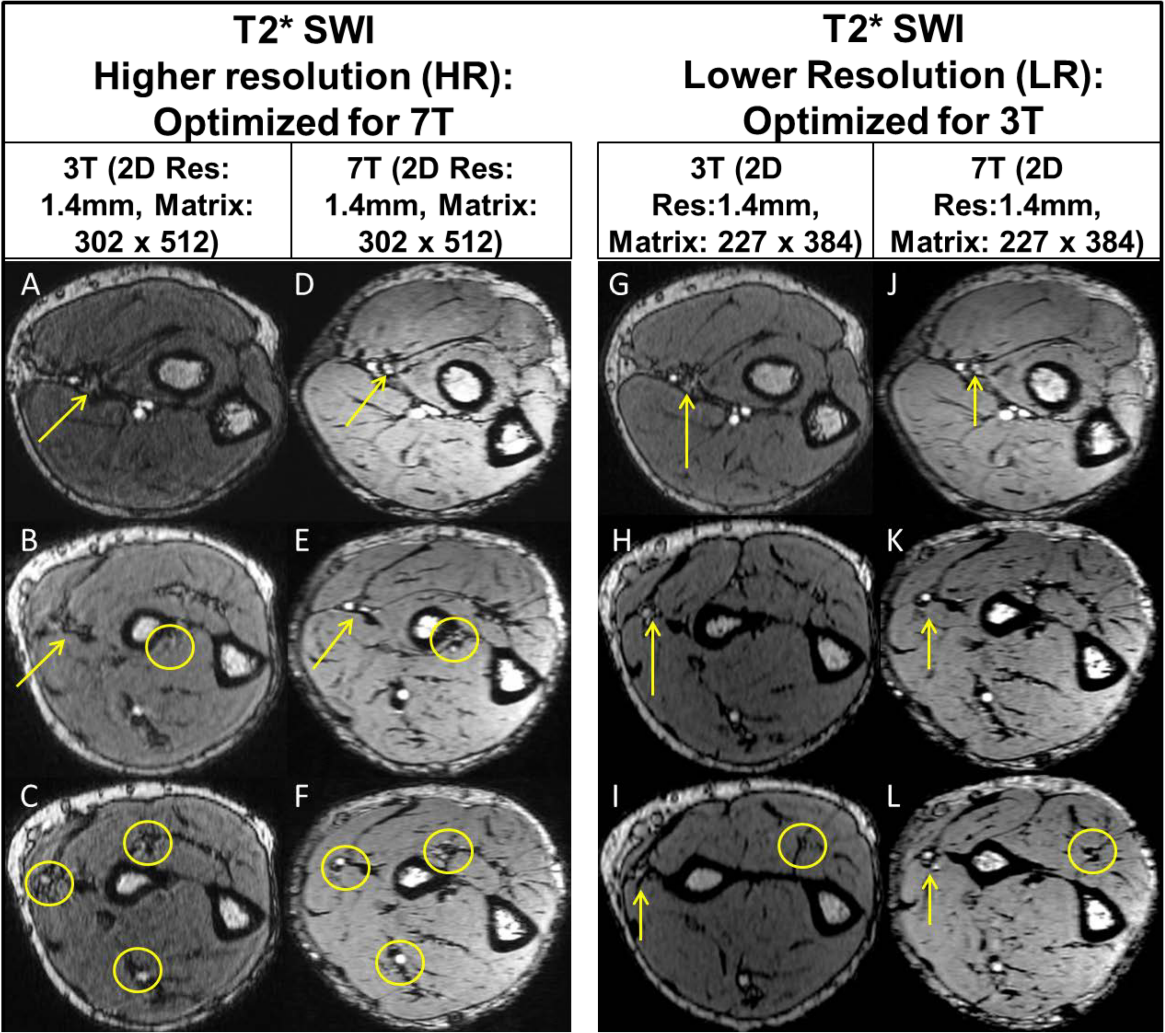
3T/7T T2* SWI: Intramuscular vascular branches [off the radial artery] are shown on the HR/LR 3T [A, B, C, G, H and I] images, but are more delineated on 7T [D, E, F, J, K and L] images. Yellow arrow and circles: indicate micro-vessel branches. Out of all three volunteer, the above data is from single volunteer only.

**Fig 5:**
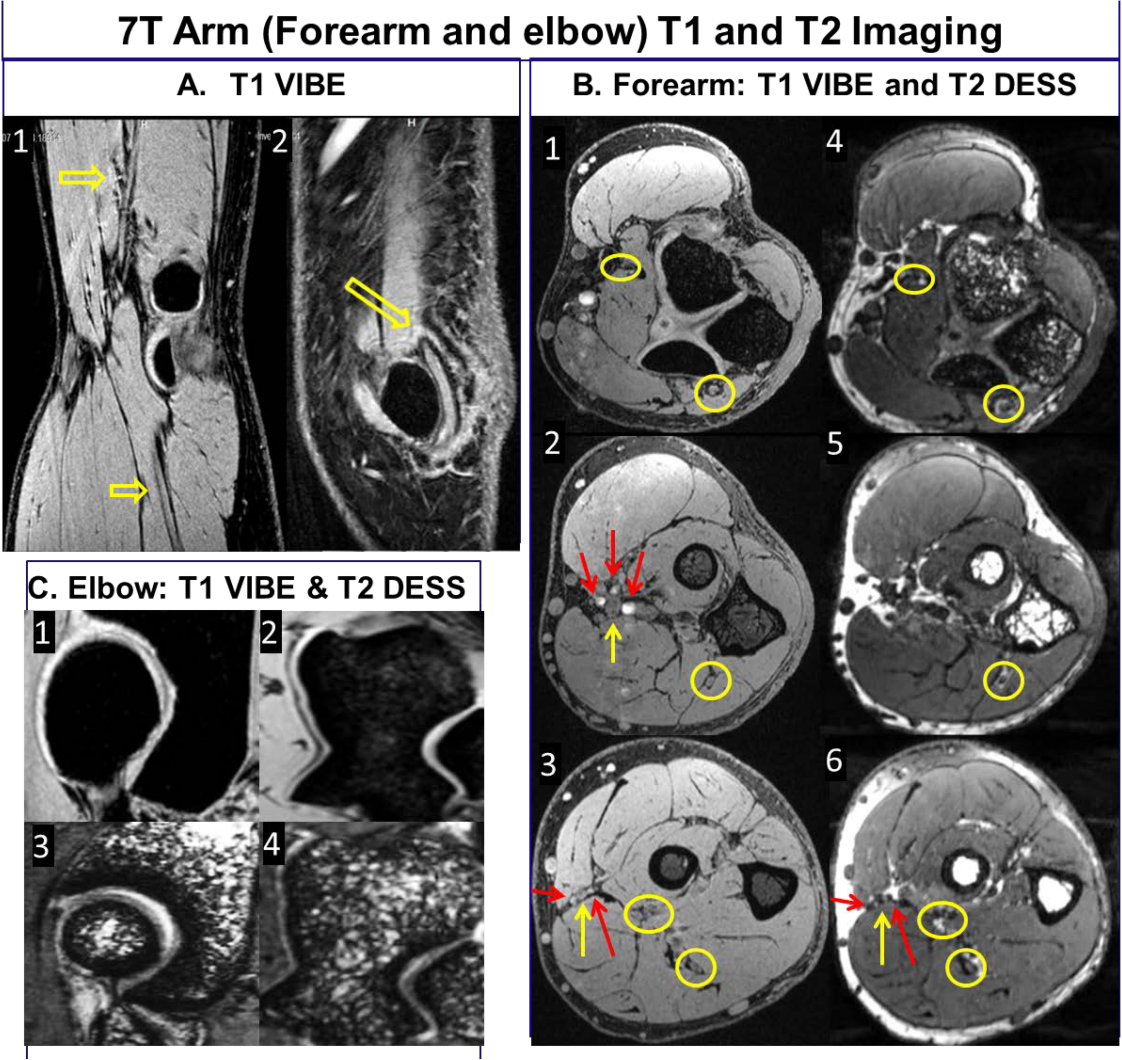
7T T1 VIBE and T2 DESS: A1 [T1 VIBE] represents radial and median nerve delineation [arrows]; A2 [T2 DESS] shows ulnar nerve [arrow]; B:1–3 [T1 VIBE] and corresponding T2 DESS [B:4–6] show three axial slices [close to elbow, forearm, and close to wrist] that depict radial, median and ulnar nerves [yellow arrows, circles] in addition to the major arteries [red arrows]; Note that there are motion and pulsatile flow artifacts [band of bright points] from median artery in anterior to posterior phase encoding direction; C:1–2 [T1 VIBE] and C:3–4 [T2 DESS] show joint anatomy depicting bone, cartilage, and synovial fluid.

**Fig 6:**
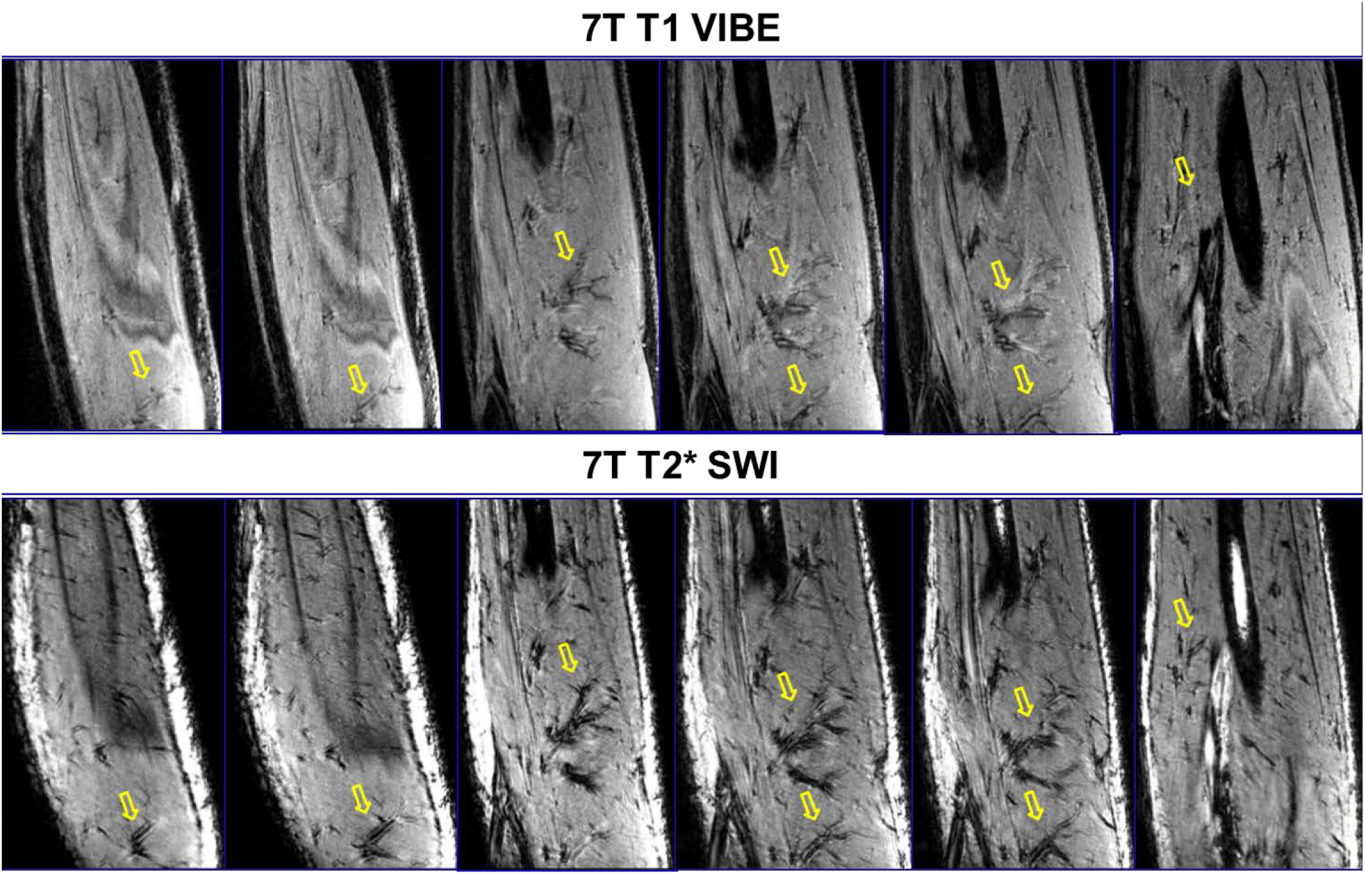
7T T2* SWI [bottom row] show marked enhancement of vascular patterns of arteries, veins and muscular perforators [marked with yellow arrow] when compared to the T1 VIBE images [top row].

#### MR image processing

**Fig 7:**
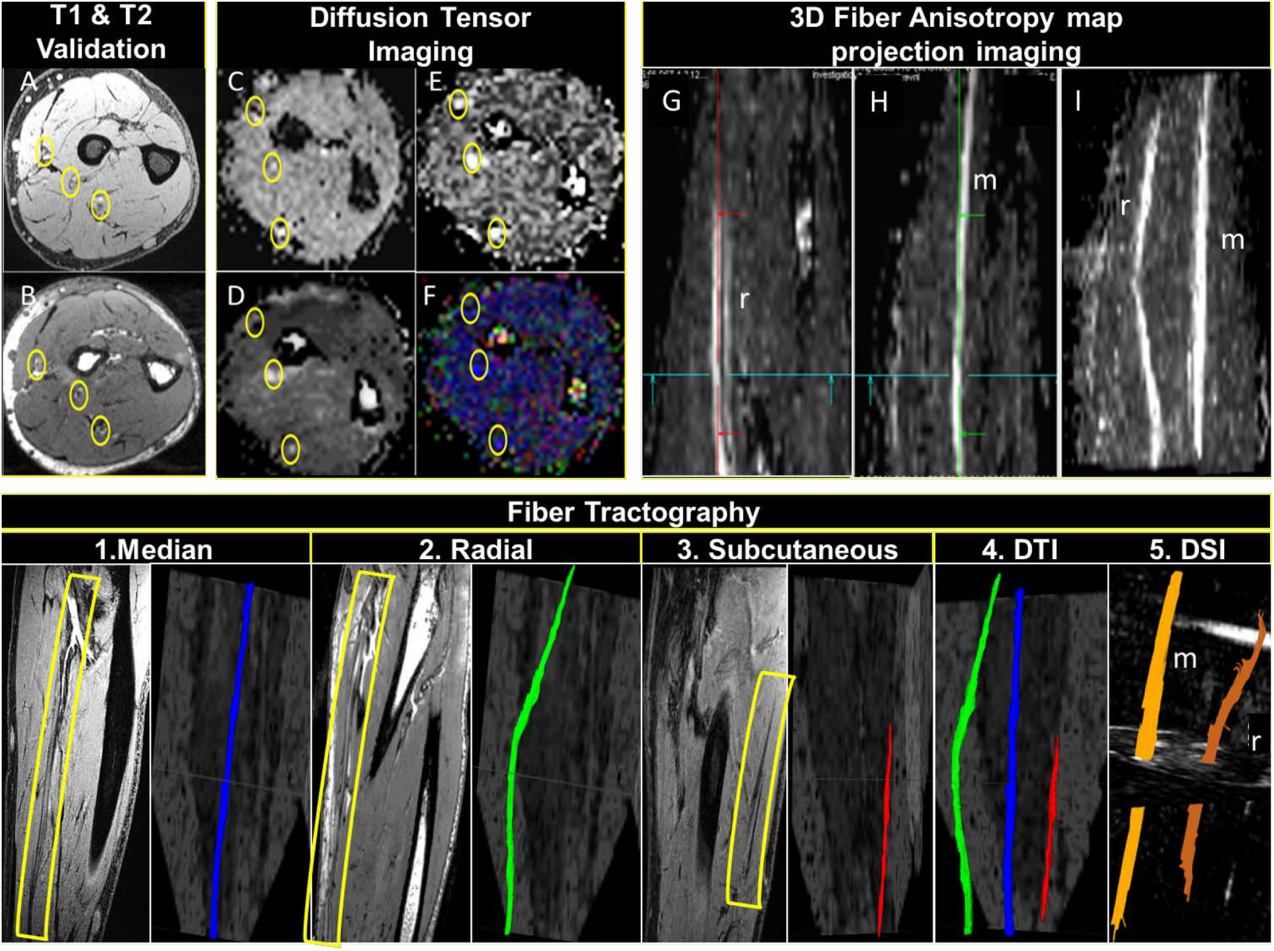
Top row: 7T DTI with T1 VIBE and T2 DESS validation: A represents T1 VIBE image and B represents T2 DESS image for validation of the locations of the forearm nerves [yellow circles]. C, D, E and F represent ADC, FA, TRACEW, and color-coded DTI map identifying forearm nerves, respectively [note the rotation of the T1 VIBE and T2 DESS images to match the DTI ones.] G and H represent FA map in sagittal view showing median [m] and radial [r] nerves using multiple intensity projection [MIP]. I demonstrates multiple intensity projection [MIP] of combined m and r nerves. Bottom row: 7T fiber tractography: 1, 2 and 3 show DTI FA maps demonstrating separate forearm nerves with anatomical validation [yellow rectangles] side by side. 4 and 5 demonstrate 3D DTI and DSI rendering showing forearm nerves tractography, respectively.

For post-processing, diffusion-weighted images were used to perform fiber tractography of peripheral nerves in forearm, and the principal fiber directions were used to conduct streamline fiber tracking. After DTI data were transferred to a local computer, FSL [FMRIB, Oxford, UK] and DSI studio [CMU, Pittsburgh, PA] were used for distortion correction and fiber tracking, respectively. Eddy current induces geometric distortion in different acquisition directions. This eddy current is produced in conductive materials/parts of RF coil by MRI gradient coils [20, 36]. Combined with subject/patient motion, it can severely damage the diffusion estimation due to pixel mismatch in the temporal image series [20]. Using eddy-correct library available in FSL [37], eddy current and motion correction were implemented where input reference image was non-diffusion weighted image [b = 0]. Then, T1 and T2 weighted images with DTI derived maps [Fig 7C–7F as later discussed in the Results Section] were utilized to locate the forearm nerves. Circular regions of interest [ROIs] were placed in the anatomic location of the nerves from the FA [Fig 7D] and color-coded maps [Fig 7F].

The fiber trajectories generated by an orientation distribution function–streamlined version of the Fiber Assignment by Continuous Tracking algorithm [38–40] were used to sample the FA and apparent diffusion coefficient [ADC] maps. Using a random seeding approach, we initiated tracking, from each random position within the seed mask, in the direction of the most prominent fiber. The Following parameters were optimized in order to distinguish nerves from surrounding various anatomical structures: an anisotropy threshold of 0.25, a step size of 0.35 to 0.5 mm, minimum fiber length of 0 to 20 mm, and a turning angle threshold of 60 degrees. The tracking was terminated when the relative fractional anisotropy for the incoming direction dropped below a preset threshold ~0.2 [heavily dependent on SNR of the specific subject’s scan data] or the turning angle exceeded 60 degrees. Each trajectory generates one profile, and all profiles were averaged to obtain the trend of the index along the fiber orientation. After anatomic confirmation of the nerve tract, mean FA and ADC values were calculated as discussed later in results section.

We used non-contrast enhanced [nCE] angiographic imaging and vessel segmentation techniques to image the digital proper palmar arteries at 7T. In order to extract the three dimensional structure of vasculature in forearm, the T1 VIBE DICOM images were exported in DICOM format to MIPAV [41]. A mask was created manually using paint grow segmentation method in MIPAV, which utilizes the concept of voxel aggregation by grouping the seed points within the volume of interest [selected manually by minimum intensity projection [MIP]]. The vasculature structures were manually traced in order to avoid errors in identifying the arteries and venous structure. After masking each segment [without skeletonizing or dilating to preserve structural vessel information], a surface and texture volume-rendering module was used to enable 3D visualization of the anatomy and segmented structures.

## Results

### 3T vs 7T

#### Quantitative analysis

Figs 1 and 2 demonstrate quantitative SNR and CNR measurements over the complete volume of forearm in T1 VIBE and T2 DESS images. For the HR protocol, the minimum SNR gain at 7T is ~ 1.8 [T1 VIBE] and ~ 2.2 [T2 DESS] times that of 3T; and the minimum CNR gain at 7T is ~ 2.2 [T1 VIBE] and ~ 2.2 [T2DESS] times that of 3T. For the LR protocol, the minimum SNR gain at 7T is ~ 1.4 [T1 VIBE] and ~ 1.8 [T2 DESS] times that of 3T; and the minimum CNR gain at 7T is ~ 1.3 [T1 VIBE] and ~ 1.7 [T2 DESS] times that of 3T. As slices close to dorsal part of forearm do not have anatomic structures suitable for measuring CNR, some slices were excluded.

In terms of RF homogeneity [a significant issue [6] at 7T], T1 VIBE and T2 DESS images [Fig 3] demonstrate uniform excitation in axial, coronal and sagittal planes covering the complete volume of forearm. On the 1^st^ subject, the uniformity was optimized by iteratively fine tuning the RF Tx coil utilizing the experimentally measured B_1_^+^ field inside the coil’s volume of interest. That tune was then utilized for all the remaining subjects. The B_1_^+^ field homogeneity demonstrated by COV [Coefficient of variation] was calculated to be 21% in the forearm [encapsulated within the coil’s volume] [26]. Also, IEC guideline was followed and SAR was numerically calculated using in-house FDTD method [6, 33]. Based on a continuous 1.97μT [averaged over the volume of the arm inside the TEM coil], the average SAR is 2.02 W/Kg and peak SAR is 8.98 W/Kg/10g [26] as IEC 60601-2-33 specifically states that MRI systems must limit locally deposited RF power to under 20 W/kg/10g. In order to demonstrate the comprehensiveness of 7T RF coil system in upper extremity imaging, the volunteers were scanned in three different regions: hand, forearm and elbow.

#### Qualitative analysis

Figs 1, 2 and 4 compare three sequences [T1 VIBE, T2 DESS and T2* SWI] for 3T vs 7T for two cases: i] HR [Optimized for 7T] and ii] LR [Optimized for 3T]. Resolution of small forearm vessels and nerves in both the superficial and deep soft tissues at HR and LR 7T imaging is shown and compared to 3T. Delineation of muscle fibers/fascial plane interfaces as well as cortical bone/soft tissue interfaces is enhanced at 7T illustrating the benefit of higher CNR.

Image Quality and artifacts evaluation: Table 3 lists all the result of qualitative analysis for image quality and artifacts. Overall image quality of T1, T2 and T2* images at 7T is higher when compared to 3T. Overall artifacts were increased from 3T to 7T but did not impact the image quality.

Fig 5 shows high resolution forearm and elbow T1 VIBE and T2 DESS 7T images. Elbow images show median, radial and ulnar nerves as smaller branches of median nerves can be seen. Three T1 and T2 comparison images [close to the hand, middle of forearm, and elbow] show various anatomical structures. These include fine delineated fascial planes, nerves fiber bundles, major arteries and smaller branches, fine cartilagenous boundaries with bright signal showing synovial fluid in between, and joint anatomy and bones [radiohumeral with trabecular detail and trochlea of ulna in the forearm].

Fig 6 displays SWI [42] images which show macro and micro vessels in addition to vessel wall without the use of any kind of Contrast dye/liquid. In comparison to the T1 VIBE slices in the top row, SWI in bottom row clearly depicts arterial vascular pattern and muscular perforators highlighting the importance of SWI at ultra-high fields.

Fig 7 demonstrates forearm nerves where 3D volume rendering and fiber tractography are shown. The quantitative track analysis results are shown in Table 4. The hand and forearm nerves correspond to known anatomic distribution of the radial, ulnar and median nerves.

**Table 4:** Quantitative DTI track analysis values for forearm nerves as shown in Fig 7, [FA: Fiber Anisotropy, ADC: Apparent diffusion coefficient, Ax: Axial/Longitudinal, Diff: Diffusivity, sd: standard deviation, and Rad: Radial].

Tract	Nerve 3	RN_Nerve1	MN_Nerve2
FA mean	0.41	0.79	0.62
FA sd	0.07	-	0.12
ADC mean	0.95	0.95	0.92
ADC sd	0.15	0.25	0.19
Ax_diff_mean	1.33	1.59	1.53
Ax_diff_sd	0.19	0.63	0.31
Rad_diff_mean	0.71	0.57	0.59
Rad_diff_sd	0.12	0.10	0.19

**Fig 8:**
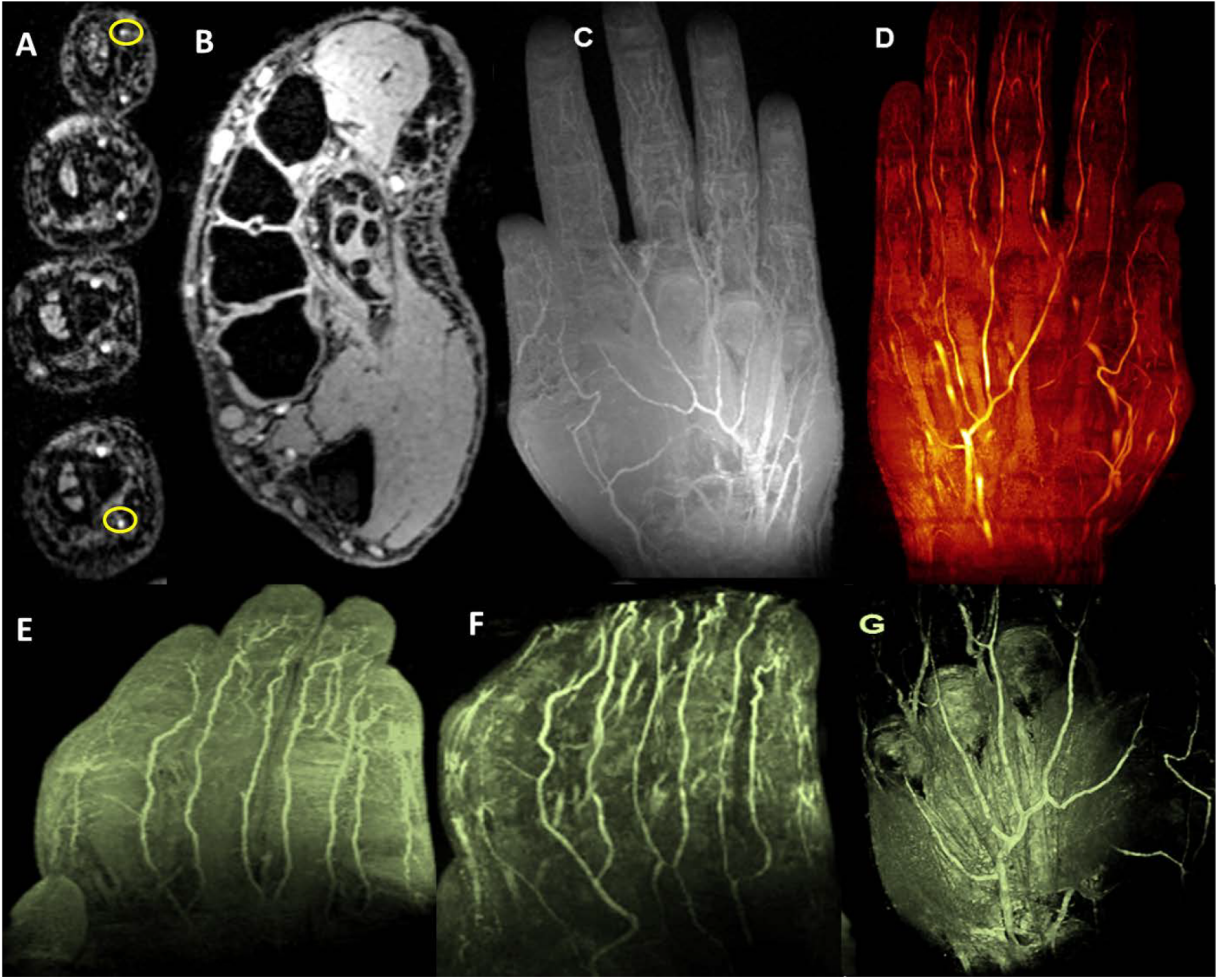
7T TOF and T1 VIBE: A [TOF] demonstrates eight proper palmar digital arteries [two of them are yellow circled], digital tendons and synovial sheaths on axial view and B [T1 VIBE] shows proper palmer arteries and two proper palmer digital arteries in thumb, and cross-sectional transmetacarpal view highlighting intrinsic muscles, flexor digitorum superficialis and profundus tendons apparatus with synovial sheaths, ligamentous structures, and inter-metacarpal vasculature. C demonstrates multiple intensity projection [MIP] of hand vasculature and [D, E and F] represent non-contrast enhanced MR angiographic images of palmar and digital microvasculature in the hand. G demonstrates 3D view of volume rendering texture [VRT].

Fig 8 demonstrates not only higher anatomical structural hand and finger images but also non-contrast enhanced angiography images. The hand and finger images depict first and second order arteries [superficial palmar and deep palmar arch], smaller proper palmar digital arteries [fingers], and fine arterial branches [finger tips].

**Fig 9:**
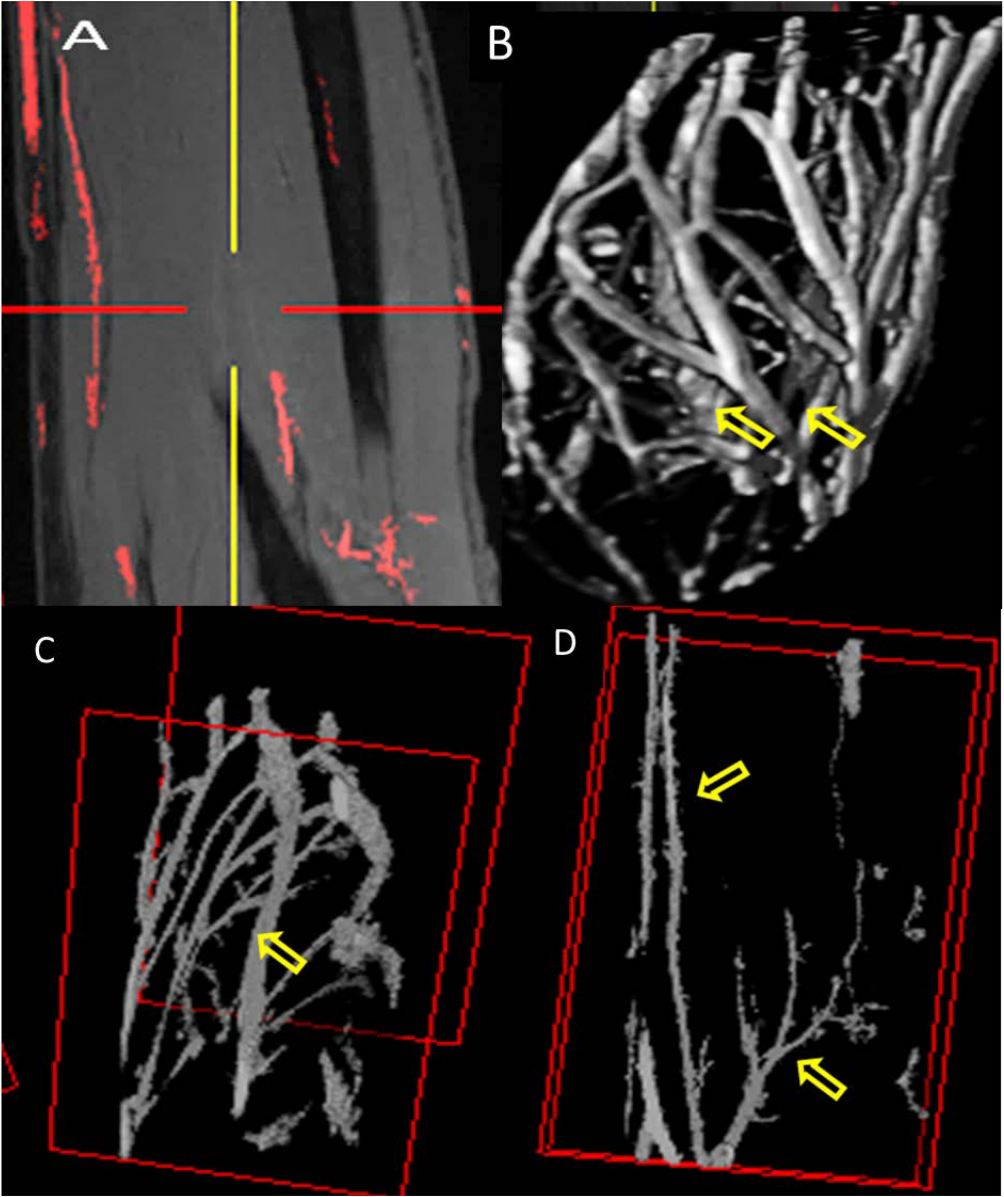
7T Vasculature Segmentation: A represents coronal image as an example of paint grows segmentation method using T1 VIBE images. B displays forearm vasculature segmentation [3D view] showing brachial artery and its branches [arrows] as well as venae comitans and superficial arteriovenous networks. C shows complete ulnar artery [arrow] and D represents radial [arrow] and brachial bifurcation [arrow] in the forearm.

Fig 9 shows the selected area of interest and the whole segmented forearm vasculature from T1 VIBE multiple intensity projection images. Fig 9 also shows the major artery [ulnar] and part of brachial bifurcation.

## Discussion

While there are no upper extremity commercial coils available at 7T [except [22]], a customized 7T RF coil was built and compared with available extremity coils at 3T with similar receive elements and comparable dimensions. The use of a combined transmit and receive coil with a small filling factor would be favorable to detect receive signals from a homogeneously excited zone of anatomy of interest. A few studies [43–46] have shown that the SNR obtained from a combined transmit and receive only system could be higher even with a limited FOV. In parallel imaging, the noise distribution is heterogeneous throughout the images. So SNR calculation is approximate but won’t be exactly accurate using ROI method [signal mean in anatomy of interest and noise standard deviation in background]. The detailed coil analysis study [47] covers the noise pre-scan method [pseudo-replica method]. When comparing a birdcage coil to a TEM coil, the current produced in end rings of the birdcage coil can generate a considerably high magnetic field component which is coaxially aligned with B_0_ static field, and therefore does not result in spin excitation. Such a component can increase Ohmic loss by inducing conduction currents in tissue [48]. Other groups have developed birdcage-based commercial coils and HEM-mode-based resonators demonstrating promising imaging capabilities limited to the wrist [22, 24, 49]. In contrast, and to our knowledge our current study is the first to explore the utility and relevance of 7T UHF MRI for the upper extremity encompassing the hand, wrist, forearm and elbow.

The quantitative analysis performed on T1 VIBE and T2 DESS images [Figs 1 and 2] demonstrates that higher SNR/CNR were achieved at 7T, almost twice when compared to 3T [most especially in the HR protocol]. As in parallel imaging, the noise distribution is heterogeneous throughout the images, therefore SNR calculation could be a crude approximation using ROI method: signal mean in anatomy of interest over standard deviation of the noise in background. Additionally, Fig 3 shows that homogenous excitation [a typical hurdle for 7T imaging] is quite possible for the forearm with coverage that extends from wrist to elbow. As a part of the qualitative analysis seen in Fig 1 [Left panel], T1 VIBE images at 7T have less overall noise when compared to the 3T images as the conspicuity of small vessels is superior. T2 DESS imaging [Fig 2 [Left panel] shows improvement in nerve signal, and better contrast between muscle planes and visualization of finer osseous trabecular detail at 7T when compared to 3T. In Fig 4, there is greater contrast and delineation of cortical bone, and improved detection of small vessel detail at 7T. Such high resolution achieved with 7T is clearly not useful when utilizing 3T as demonstrated in Figs 1, 2 and 4.

Diffusion-based MRI is utilized as a non-invasive, non-disruptive strategy for sequential assessment of forearm nerves. It is quantified by water diffusion parameters [FA and ADC] as indirect correlates or surrogates of neuroregeneration after transection repair or transplant related nerve outcomes. In order to increase the reliability and interpretation of results, DTI derived maps [FA, ADC, and color-coded] were defined in conjunction with T1 and T2 anatomical validation. High resolution imaging of peripheral nerves may have application in the detection of neuropathy and monitoring to assess disease progression or response to appropriate therapy [15, 30] [50]. Moreover, volume rendering and 3D depiction of the course of the forearm nerves [as shown Fig 7] may prove useful in the broader realm of reconstructive surgical or vascularized composite allotransplantation [VCA] applications. While comparison to 3T [in terms of DTI] was outside of scope for this study, the presented 7T images show very good delineation of the forearm nerves, without the use of contrast agents.

7T TOF imaging [shown Fig 8] and corresponding vascular segmentation [shown in Fig 9] allow depiction of macro and microvascular anatomy of the hand, forearm, and hand [wrist] in 2D monitoring of microvascular integrity as it relates to certain vascular disease processes [32, 51]. In addition, SWI [Fig 6] could be especially useful in visualizing various macro or micro-vascular pathologies secondary to vascular trauma, thrombotic/embolic occlusion, and neovascularization of tumors, and in high-resolution MR venography [19, 32, 52]. While we optimized the SWI sequence for image quality, this specific sequence with the current standard clinical practices, may not be appropriate for studying the anatomic structures in question. While contrast enhanced imaging is utilized extensively [53], the fact that 7T high quality vascular imaging can be accomplished without the use of intravenous contrast is invaluable in patients with organ transplantation, autoimmune vasculitis or diabetic peripheral vascular disease as these conditions often have concomitant renal vasculitis and/or renal insufficiency.

MR angiography using 1.5T imaging has been previously described in evaluating neuropathic leg pain [54] and other diabetic vasculopathy [55]. 3T imaging has been reported in evaluation of the brachial plexus [56, 57]. In addition, 3T MR angiography has also been used in imaging of intracranial vessel disease/vasculitis [58, 59]. 7T MR angiography has also been explored [60] specifically in the upper extremity focusing on wrist [61] or hand [49] but limited to the palmar vasculature. The work presented here expands higher resolution MRA to other regions including the digital vasculature and capillary networks in the pulps of fingers.

The choice of CT Angiography [CTA] vs MRA or vice versa for specific indications is still a debatable issue [62–64]. The choice of CTA, usually depends upon the region of interest involved in either neuro [65–68], coronary [34, 69–72] or rest of the body [67, 73] related applications. However, the merits of MRA over CTA cannot be ignored especially in the case of UHF 7T MRA. Work in 7T TOF, nCE MRA, SWI, and other MR based methods [DCE_MRA, MRV, DTI, etc.] could open new possibilities for nCE vascular imaging as confirmed by our study and validated by other research studies [19, 60, 73–77]. While CTA faces contrast-agent related nephrotoxic or anaphylotoxic risks, it has the advantage of a faster scan time that could be important in emergent clinical indications such as pulmonary embolism or acute cerebrovascular disease [65]. Earlier work however has shown that the diagnostic performance of 3T CE whole-heart coronary MR angiography approaches the diagnostic performance of 64-section CT [78]. In addition, 7T UHF MRI, and parallel imaging with higher number of receive channels could produce highly improved signal/contrast to noise ratio, faster scanning time, and significant advantage for repeated or longitudinal/sequential imaging applications [78–80]. Furthermore, the avoidance of radiation by MR offers critical advantage in pediatric imaging especially of the craniofacial skeleton [in concussion or traumatic brain injury] [81]. When compared to MR related research studies, a significant growth in research utilization of CT has been observed [82]. However, there is need for more MRA studies [especially utilizing UHF MRI] [19, 60, 73–77]. Non-invasive methods like nCE MRA, TOF, SWI, and diffusion related methods have very high potential in safe, non-radiation, nCE detection of high-signal-intensity plaque, coronary arterial wall [69], and atherosclerotic vascular disease [83, 84] as well as in a host of other indications [19, 60, 65–70, 73, 74, 85]. UHF 7T imaging can alleviate and overcome several imaging challenges encountered in neurovascular disease at lower field strengths [1.5T and 3T] like venous contamination obscuring underlying arterial architecture or delineation of contextual anatomical structures [65].

## Conclusion

High quality non-contrast enhanced ultra-high resolution neuro-vascular upper extremity imaging is possible at 7T. High quality 7T images were obtained using T1 VIBE, T2 DESS and T2* SWI sequences as well as TOF, DTI, and DSI sequences in conjunction with a custom-designed RF coil system. Analysis demonstrate that 1] CNR/SNR at 7T are almost two folds [for T1 VIBE and T2 DESS sequences] of that achieved at 3T for upper extremity applications and 2] broader clinical potential is present for 7T imaging in musculoskeletal applications.
